# The chromosomal genome sequence of the moon jellyfish, *Aurelia* sp. 4 Dawson *et al*. 2005 (Semaeostomeae: Ulmaridae) and its associated microbial metagenome sequences

**DOI:** 10.12688/wellcomeopenres.25907.1

**Published:** 2026-03-25

**Authors:** Michael N. Dawson, Graeme Oatley, Elizabeth Sinclair, Eerik Aunin, Noah Gettle, Camilla Santos, Michael Paulini, Haoyu Niu, Victoria McKenna, Rebecca O’Brien, Wellcome Sanger Institute Tree of Life Management, Samples and Laboratory team, Wellcome Sanger Institute Scientific Operations: Sequencing Operations, Wellcome Sanger Institute Tree of Life Core Informatics team, EBI Aquatic Symbiosis Genomics Data Portal Team, Aquatic Symbiosis Genomics Project Leadership

**Affiliations:** 1 University of California Merced Dawson Lab, Merced, California, USA; 2 Tree of Life Programme, Wellcome Sanger Institute, Hinxton, England, UK

**Keywords:** Aurelia sp. 4 Dawson et al. 2005; moon jellyfish; genome sequence; chromosomal; Semaeostomeae; microbial metagenome assembly

## Abstract

We present a genome assembly from an individual *Aurelia* sp. 4 Dawson *et al*., 2005 (moon jellyfish; Cnidaria; Scyphozoa; Semaeostomeae; Ulmaridae). The genome sequence has a total length of 462.10 megabases. Most of the assembly (99.99%) is scaffolded into 21 chromosomal pseudomolecules. The mitochondrial genome has also been assembled, with a length of 16.88 kilobases. From the metagenome data, we recovered 3 bins, of which 2 were high-quality MAGs.

## Species taxonomy

Eukaryota; Opisthokonta; Metazoa; Eumetazoa; Cnidaria; Scyphozoa; Semaeostomeae; Ulmaridae; *Aurelia*; unclassified *Aurelia*; *Aurelia* sp. 4 Dawson *et al*. (2005) (NCBI:txid2790877).

## Background

The moon jellyfish, *Aurelia*, is the only scyphozoan jellyfish that visibly resembles its eponymous celestial body. Ghostly white, mostly, though other times pink (a ‘blood moon’?), purplish, electric blue, they float or swim in the near-surface coastal waters of the world’s oceans and seas. Distinguishing one species of *Aurelia* from another by appearance alone is, however, difficult. The use of DNA sequence has become a routine complement to quantitative morphological characteristics in species identification and delimitation ( Dawson, 2003; Gómez-Daglio & Dawson, 2017; Scorrano *et al.*, 2017).

This synthesis of molecular and morphological data in ‘integrative taxonomy’ is helping to stabilise the systematics of the genus, allowing the ecological and distributional information that exists to be better assigned to particular species ( Abboud *et al.*, 2018). However, the vast majority of species remain an enigma. One such example is *Aurelia* sp. 4, which is known best from marine lakes (landlocked bodies of seawater) in the western Pacific archipelago of Palau. The species is better studied than many congeners ( Dawson, 2003; Dawson *et al.*, 2005; Hamner *et al.*, 1982; Martin, 1999). For example, we know from the location of both polyps and medusae that its life history is completed within a marine lake and we also know in some detail its diel vertical migration and diet ( Hamner *et al.*, 1982; Martin, 1999). But this information is not available elsewhere in the species’ range, for example in marine lakes in Borneo or anthropogenic habitat in Hawai’i. Thus, intriguing biological questions remain to be resolved, such as the nature of morphological variation among populations, which surpasses that among some species ( Dawson, 2003), details of genetic variation and the extent of local adaptation among ( Dawson & Martin, 2001), and from where it was most likely introduced into Hawai’i ( Dawson *et al.*, 2005).

Nonetheless, as for other species of *Aurelia*, we do know with certainty – confirmed by working occasionally with these animals in the field since the mid-1990s – that they do not form a photosymbiosis with zooxanthellae. As such, *Aurelia* sp. 4 was chosen for the Aquatic Symbiosis Genomics project to provide a contrast with the zooxanthellate jellyfishes (e.g. in behaviours such as diel horizontal and vertical migration), a comparison between benthic and pelagic forms (i.e. polyp vs medusa), and to gain a sense of how the small spatial scale of variation in marine lake environments and genetics interact to shape associations with non-photosymbiotic co-bionts (e.g. Tinta *et al.*, 2012).

While *Aurelia* is generally thought likely to benefit from anthropogenic environmental change, populations of *Aurelia* sp.4, more than other species, might be considered at risk of negative outcomes. Their isolated marine lake habitats have been prone to large environmental fluctuations (e.g. Martin *et al.*, 2006) and in the past several decades what once were considered perennial populations of moon jellyfish medusae have at times been absent (pers. obs.). It is fortunate, therefore, that the marine lakes are recognized as a key component deserving protection under the auspices of the UNESCO World Heritage site in Palau ([ https://whc.unesco.org/en/list/1386]) though whether such designation can buffer against climate change is another matter.

Multiple reference genome sequences now exist for the genus, including one from California PRJNA490213 ( Gold *et al.*, 2019), another from the Baltic Sea representing the type species *A. aurita* (Linnaeus, 1758) PRJNA494057 ( Khalturin *et al.*, 2019), and a third from a widely used laboratory strain originating in the Pacific Ocean PRJNA494062 ( Khalturin *et al.*, 2019). To this, we add *Aurelia* sp. 4 and, in a companion paper, *Aurelia* sp. 3, also from Palau (PRJEB74053; in preparation).

As has been the case for many marine taxa, *Aurelia* has historically been considered to possess little geographic variation, even being considered to have a single, ubiquitous, circumglobal, generalist species. By providing another high quality reference genome for this iconic genus, we enhance the potential for understanding the dynamics of genome evolution at a range of fine spatial, temporal, and taxonomic scales, relative to the higher taxonomic levels that previously have been possible (e,g, Nong *et al.*, 2020). This unprecedented resolution promises detailed insight into the genomic underpinnings of scyphozoans that are and are not photosymbiotic.

## Methods

### Sample acquisition

**Figure 1.**
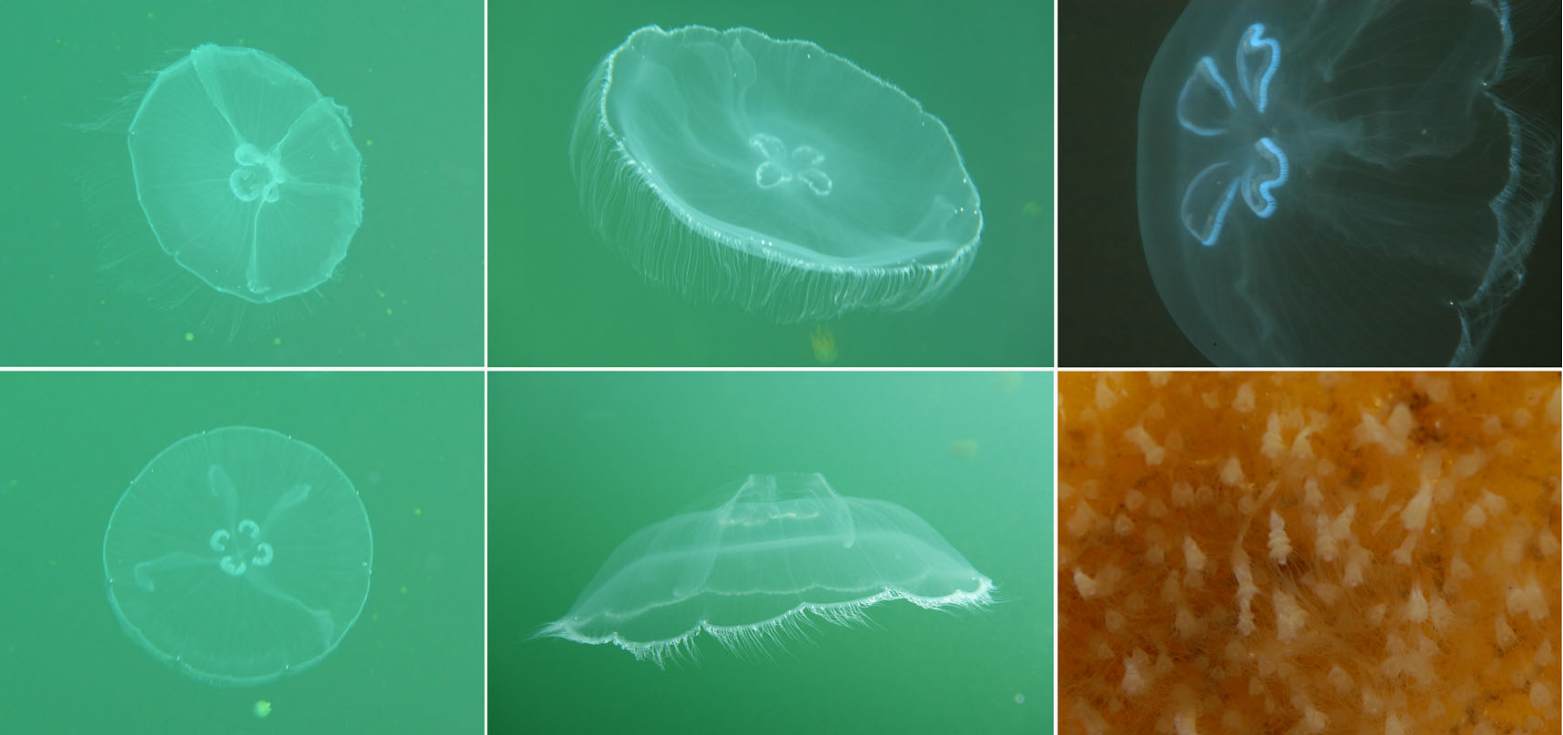
*Aurelia* sp. 4 from three marine lakes in Palau. (Left) Medusae from Uet era Ngermeuangel, Koror. (Centre) Medusae from Ongeim’l Tketau, also known as “Jellyfish Lake”. (Right) Medusa and polyps from T-Lake, a.k.a. “Lake Ten” ( Hamner & Hamner, 1998). Photographs by (left, centre) Laura E. Martin and (right) Lori J. Bell.

Specimens were collected by freedivers using large plastic bags. Live specimens were transported to the Coral Reef Research Foundation (Koror, Palau), where tissues were biopsied and immediately snap-frozen in liquid nitrogen. Samples were stored and shipped in an ultra-cold dry shipper and, on receipt at the University of California, Merced, transferred to a −80 °C freezer until forwarding to the Wellcome Sanger Institute on dry ice. The specimen used for genome sequencing was an adult *Aurelia* sp. 4 (specimen ID UCALI0000022, ToLID jsAurSpec1; Figure 1), collected from Ongeim’L Tketau, Koror, Palau (latitude 7.161, longitude 134.3764) on 2022-01-08. The specimen was collected and identified by Michael Dawson. The same specimen was used for RNA sequencing.

### Nucleic acid extraction

Protocols for high molecular weight (HMW) DNA extraction developed at the Wellcome Sanger Institute (WSI) Tree of Life Core Laboratory are available on protocols.io ( Howard *et al.*, 2025). The jsAurSpec1 sample was weighed and triaged to determine the appropriate extraction protocol. Tissue from the **other somatic animal tissue** was homogenised by powermashing using a PowerMasher II tissue disruptor. HMW DNA was extracted using the Modified Omega Biotek protocol. We used centrifuge-mediated fragmentation to produce DNA fragments in the 8–10 kb range, following the Covaris g-TUBE protocol for ultra-low input (ULI). Sheared DNA was purified by automated SPRI (solid-phase reversible immobilisation). The concentration of the sheared and purified DNA was assessed using a Nanodrop spectrophotometer and Qubit Fluorometer using the Qubit dsDNA High Sensitivity Assay kit. Fragment size distribution was evaluated by running the sample on the FemtoPulse system.

RNA was extracted from tissue of jsAurSpec1 in the Tree of Life Laboratory at the WSI using the RNA Extraction: Automated MagMax™ *mir*Vana protocol. The RNA concentration was assessed using a Nanodrop spectrophotometer and a Qubit Fluorometer using the Qubit RNA Broad-Range Assay kit. Analysis of the integrity of the RNA was done using the Agilent RNA 6000 Pico Kit and Eukaryotic Total RNA assay.

### PacBio HiFi library preparation and sequencing

Library preparation and sequencing were performed at the WSI Scientific Operations core. Prior to library preparation, the DNA was fragmented to ~10 kb. Ultra-low-input (ULI) libraries were prepared using the PacBio SMRTbell® Express Template Prep Kit 2.0 and gDNA Sample Amplification Kit. Samples were normalised to 20 ng DNA. Single-strand overhang removal, DNA damage repair, and end-repair/A-tailing were performed according to the manufacturer’s instructions, followed by adapter ligation. A 0.85× pre-PCR clean-up was carried out with Promega ProNex beads.

The DNA was evenly divided into two aliquots for dual PCR (reactions A and B), both following the manufacturer’s protocol. A 0.85× post-PCR clean-up was performed with ProNex beads. DNA concentration was measured using a Qubit Fluorometer v4.0 (Thermo Fisher Scientific) with the Qubit HS Assay Kit, and fragment size was assessed on an Agilent Femto Pulse Automated Pulsed Field CE Instrument (Agilent Technologies) using the gDNA 55 kb BAC analysis kit. PCR reactions A and B were then pooled, ensuring a total mass of ≥500 ng in 47.4 μl.

The pooled sample underwent another round of DNA damage repair, end-repair/A-tailing, and hairpin adapter ligation. A 1× clean-up was performed with ProNex beads, followed by DNA quantification using the Qubit and fragment size analysis using the Agilent Femto Pulse. Size selection was performed on the Sage Sciences PippinHT system, with target fragment size determined by Femto Pulse analysis (typically 4–9 kb). Size-selected libraries were cleaned with 1.0× ProNex beads and normalised to 2 nM before sequencing.

The sample was sequenced on a Revio instrument (Pacific Biosciences). The prepared library was normalised to 2 nM, and 15 μL was used for making complexes. Primers were annealed and polymerases bound to generate circularised complexes, following the manufacturer’s instructions. Complexes were purified using 1.2X SMRTbell beads, then diluted to the Revio loading concentration (200–300 pM) and spiked with a Revio sequencing internal control. The sample was sequenced on a Revio 25M SMRT cell. The SMRT Link software (Pacific Biosciences), a web-based workflow manager, was used to configure and monitor the run and to carry out primary and secondary data analysis.

### Hi-C

***Sample preparation and crosslinking***

The Hi-C sample was prepared from 20–50 mg of frozen tissue from the intestine of the jsAurSpec1 sample using the Arima-HiC v2 kit (Arima Genomics). Following the manufacturer’s instructions, tissue was fixed and DNA crosslinked using TC buffer to a final formaldehyde concentration of 2%. The tissue was homogenised using the Diagnocine Power Masher-II. Crosslinked DNA was digested with a restriction enzyme master mix, biotinylated, and ligated. Clean-up was performed with SPRISelect beads before library preparation. DNA concentration was measured with the Qubit Fluorometer (Thermo Fisher Scientific) and Qubit HS Assay Kit. The biotinylation percentage was estimated using the Arima-HiC v2 QC beads.

***Hi-C library preparation and sequencing***

Biotinylated DNA constructs were fragmented using a Covaris E220 sonicator and size selected to 400–600 bp using SPRISelect beads. DNA was enriched with Arima-HiC v2 kit Enrichment beads. End repair, A-tailing, and adapter ligation were carried out with the NEBNext Ultra II DNA Library Prep Kit (New England Biolabs), following a modified protocol where library preparation occurs while DNA remains bound to the Enrichment beads. Library amplification was performed using KAPA HiFi HotStart mix and a custom Unique Dual Index (UDI) barcode set (Integrated DNA Technologies). Depending on sample concentration and biotinylation percentage determined at the crosslinking stage, libraries were amplified with 10–16 PCR cycles. Post-PCR clean-up was performed with SPRISelect beads. Libraries were quantified using the AccuClear Ultra High Sensitivity dsDNA Standards Assay Kit (Biotium) and a FLUOstar Omega plate reader (BMG Labtech).

Prior to sequencing, libraries were normalised to 10 ng/μL. Normalised libraries were quantified again to create equimolar and/or weighted 2.8 nM pools. Pool concentrations were checked using the Agilent 4200 TapeStation (Agilent) with High Sensitivity D500 reagents before sequencing. Sequencing was performed using paired-end 150 bp reads on the Illumina NovaSeq X.

### RNA library preparation and sequencing

Libraries were prepared using the NEBNext ^®^ Ultra™ II Directional RNA Library Prep Kit for Illumina (New England Biolabs), following the manufacturer’s instructions. Poly(A) mRNA in the total RNA solution was isolated using oligo(dT) beads, converted to cDNA, and uniquely indexed; 14 PCR cycles were performed. Libraries were size-selected to produce fragments between 100–300 bp. Libraries were quantified, normalised, pooled to a final concentration of 2.8 nM, and diluted to 150 pM for loading. Sequencing was carried out on the Illumina NovaSeq X, generating paired-end reads.

### Genome assembly

Prior to assembly of the PacBio HiFi reads, a database of *k*-mer counts ( *k* = 31) was generated from the filtered reads using FastK. GenomeScope2 ( Ranallo-Benavidez *et al.*, 2020) was used to analyse the *k*-mer frequency distributions, providing estimates of genome size, heterozygosity, and repeat content.

The HiFi reads were assembled using Hifiasm ( Cheng *et al.*, 2021) with the --primary option. Haplotypic duplications were identified and removed using purge_dups ( Guan *et al.*, 2020). The Hi-C reads ( Rao *et al.*, 2014) were mapped to the primary contigs using bwa-mem2 ( Vasimuddin *et al.*, 2019), and the contigs were scaffolded in YaHS ( Zhou *et al.*, 2023) with the --break option for handling potential misassemblies. The scaffolded assemblies were evaluated using Gfastats ( Formenti *et al.*, 2022), BUSCO ( Manni *et al.*, 2021) and MERQURY.FK ( Rhie *et al.*, 2020).

The mitochondrial genome was assembled using MitoHiFi ( Uliano-Silva *et al.*, 2023).

### Assembly curation

The assembly was decontaminated using the Assembly Screen for Cobionts and Contaminants ( ASCC) pipeline. TreeVal was used to generate the flat files and maps for use in curation. Manual curation was conducted primarily in PretextView and HiGlass ( Kerpedjiev *et al.*, 2018). Scaffolds were visually inspected and corrected as described by Howe *et al*. (2021). Manual corrections included 30 breaks, 41 joins, and removal of 26 haplotypic duplications. This reduced the scaffold count by 56.8% and reduced the total assembly length by 2.6%. The curation process is described at https://gitlab.com/wtsi-grit/rapid-curation . PretextSnapshot was used to generate a Hi-C contact map of the final assembly.

### Assembly quality assessment

The Merqury.FK tool ( Rhie *et al.*, 2020) was run in a Singularity container ( Kurtzer *et al.*, 2017) to evaluate *k*-mer completeness and assembly quality for the primary and alternate haplotypes using the *k*-mer databases ( *k* = 31) computed prior to genome assembly. The analysis outputs included assembly QV scores and completeness statistics.

The genome was analysed using the BlobToolKit pipeline, a Nextflow implementation of the earlier Snakemake version ( Challis *et al.*, 2020). The pipeline aligns PacBio reads using minimap2 ( Li, 2018) and SAMtools ( Danecek *et al.*, 2021) to generate coverage tracks. It runs BUSCO ( Manni *et al.*, 2021) using lineages identified from the NCBI Taxonomy ( Schoch *et al.*, 2020). For the three domain-level lineages, BUSCO genes are aligned to the UniProt Reference Proteomes database ( Bateman *et al.*, 2023) using DIAMOND blastp ( Buchfink *et al.*, 2021). The genome is divided into chunks based on the density of BUSCO genes from the closest taxonomic lineage, and each chunk is aligned to the UniProt Reference Proteomes database with DIAMOND blastx. Sequences without hits are chunked using seqtk and aligned to the NT database with blastn ( Altschul *et al.*, 1990). The BlobToolKit suite consolidates all outputs into a blobdir for visualisation. The BlobToolKit pipeline was developed using nf-core tooling ( Ewels *et al.*, 2020) and MultiQC ( Ewels *et al.*, 2016), with containerisation through Docker ( Merkel, 2014) and Singularity ( Kurtzer *et al.*, 2017).

## Metagenome assembly

The metagenome assembly was generated using MetaMDBG ( Benoit *et al.*, 2024). The resulting bin sets of each binning algorithm were optimised and refined using DAS Tool ( Sieber *et al.*, 2018). PROKKA ( Seemann, 2014) was used to identify tRNAs and rRNAs in each bin, CheckM ( Parks *et al.*, 2015) (checkM_DB release 2015-01-16) was used to assess bin completeness/contamination, and GTDB-Tk ( Chaumeil *et al.*, 2022) (GTDB release 214) was used to taxonomically classify bins. Taxonomic replicate bins were identified using dRep ( Olm *et al.*, 2017) with default settings (95% ANI threshold). All bins were assessed for quality and categorised as metagenome-assembled genomes (MAGs) if they met the following criteria: contamination ≤ 5%, presence of 5S, 16S, and 23S rRNA genes, at least 18 unique tRNAs, and either ≥ 90% completeness or ≥ 50% completeness with fully circularised chromosomes ( Bowers *et al.*, 2017). Bins that did not meet these thresholds, or were identified as taxonomic replicates of MAGs, were retained as ‘binned metagenomes’ provided they had ≥ 50% completeness and ≤ 10% contamination.

## Genome sequence report

### Sequence data

**Figure 2.**
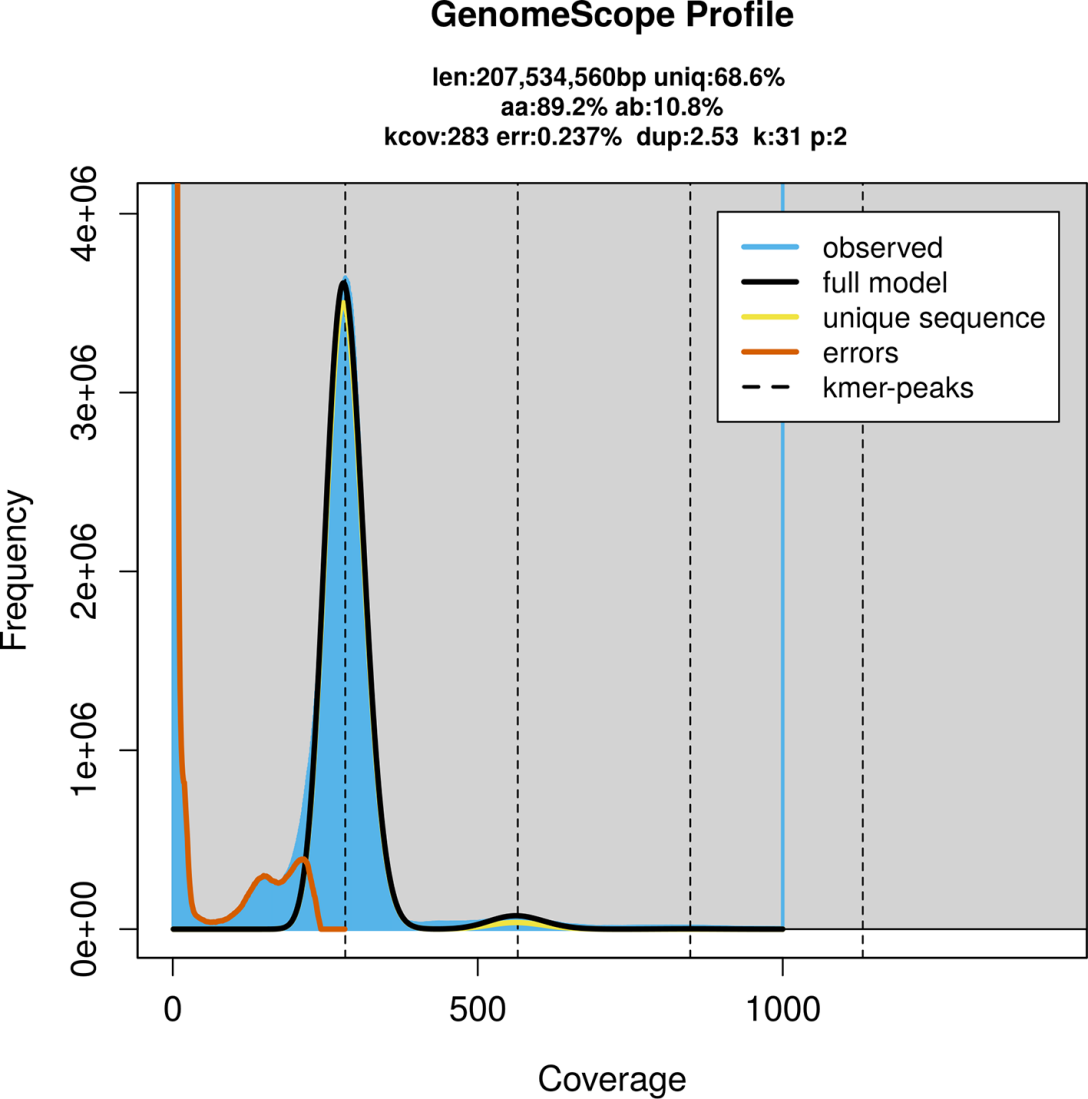
Frequency distribution of *k*-mers generated using GenomeScope2. The plot shows observed and modelled *k*-mer spectra, providing estimates of genome size, heterozygosity, and repeat content based on unassembled sequencing reads.

PacBio sequencing of the *Aurelia* sp. 4 specimen generated 128.80 Gb (gigabases) from 20.23 million reads, which were used to assemble the genome. GenomeScope2.0 analysis estimated the haploid genome size at 207.74 Mb, with a heterozygosity of 50.00% and repeat content of 31.43% ( Figure 2). These estimates guided expectations for the assembly. Based on the estimated genome size, the sequencing data provided approximately 154× coverage. Hi-C sequencing produced 159.53 Gb from 1 056.49 million reads, which were used to scaffold the assembly. RNA sequencing data were also generated and are available in public sequence repositories. Table 1 summarises the specimen and sequencing details.

**Table 1.** Specimen and sequencing data for BioProject PRJEB74055.

Platform	PacBio HiFi	Hi-C	RNA-seq
**ToLID**	jsAurSpec1	jsAurSpec1	jsAurSpec1
**Specimen ID**	UCALI0000022	UCALI0000022	UCALI0000022
**BioSample (source individual)**	SAMEA112358987	SAMEA112358987	SAMEA112358987
**BioSample (tissue)**	SAMEA112359050	SAMEA112359051	SAMEA112359046
**Instrument**	Revio	Illumina NovaSeq X	Illumina NovaSeq X
**Run accessions**	ERR12779262; ERR12804364	ERR12791490; ERR14224593	ERR13148254
**Read count total**	20.23 million	1 056.49 million	63.17 million
**Base count total**	128.80 Gb	159.53 Gb	9.54 Gb

### Assembly statistics

The primary haplotype was assembled, and contigs corresponding to an alternate haplotype were also deposited in INSDC databases. The final assembly has a total length of 462.10 Mb in 31 scaffolds, with 160 gaps, and a scaffold N50 of 21.79 Mb ( Table 2).

**Table 2.** Genome assembly statistics.

**Assembly name**	jsAurSpec1.1
**Assembly accession**	GCA_964019985.1
**Alternate haplotype accession**	GCA_964019785.1
**Assembly level**	chromosome
**Span (Mb)**	462.10
**Number of chromosomes**	21
**Number of contigs**	191
**Contig N50**	5.47 Mb
**Number of scaffolds**	31
**Scaffold N50**	21.79 Mb
**Organelles**	Mitochondrion: 16.88 kb

**Figure 3.**
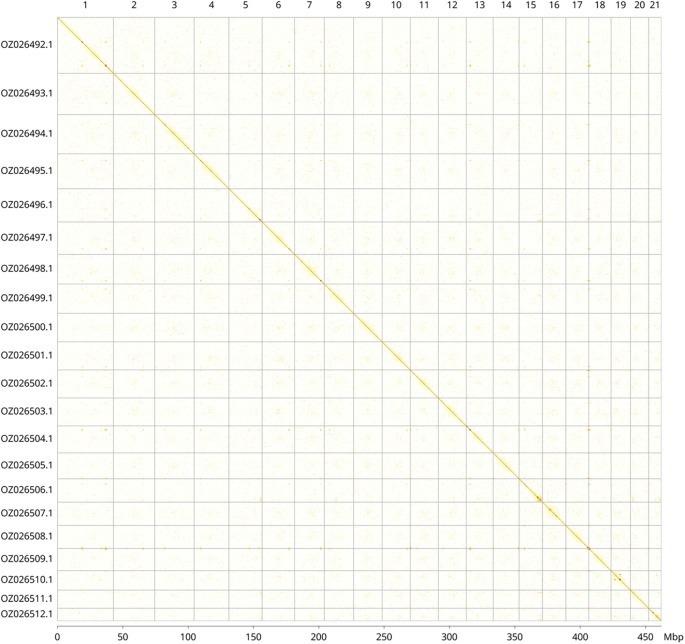
Hi-C contact map of the *Aurelia* sp. 4 genome assembly. Assembled chromosomes are shown in order of size and labelled along the axes, with a megabase scale shown below. The plot was generated using PretextSnapshot.

Most of the assembly sequence (99.99%) was assigned to 21 chromosomal-level scaffolds. These chromosome-level scaffolds, confirmed by Hi-C data, are named according to size ( Figure 3; Table 3).

**Table 3.** Chromosomal pseudomolecules in the primary genome assembly of *Aurelia* sp. 4 jsAurSpec1.

INSDC accession	Molecule	Length (Mb)	GC%
OZ026492.1	1	42.93	39.50
OZ026493.1	2	31.51	38.50
OZ026494.1	3	30.21	38.50
OZ026495.1	4	26.57	38.50
OZ026496.1	5	25.53	39
OZ026497.1	6	24.82	38.50
OZ026498.1	7	22.69	38.50
OZ026499.1	8	22.46	38.50
OZ026500.1	9	21.79	38
OZ026501.1	10	21.47	38
OZ026502.1	11	21.45	39
OZ026503.1	12	21.43	38.50
OZ026504.1	13	20.60	39
OZ026505.1	14	19.80	39
OZ026506.1	15	17.92	38.50
OZ026507.1	16	17.84	38
OZ026508.1	17	17.52	39
OZ026509.1	18	17.08	39.50
OZ026510.1	19	14.89	38.50
OZ026511.1	20	13.97	39
OZ026512.1	21	9.59	38

The mitochondrial genome was also assembled (length 16.88 kb, OZ026513.1). This sequence is included as a contig in the multifasta file of the genome submission and as a standalone record.

### Assembly quality metrics

**Figure 4.**
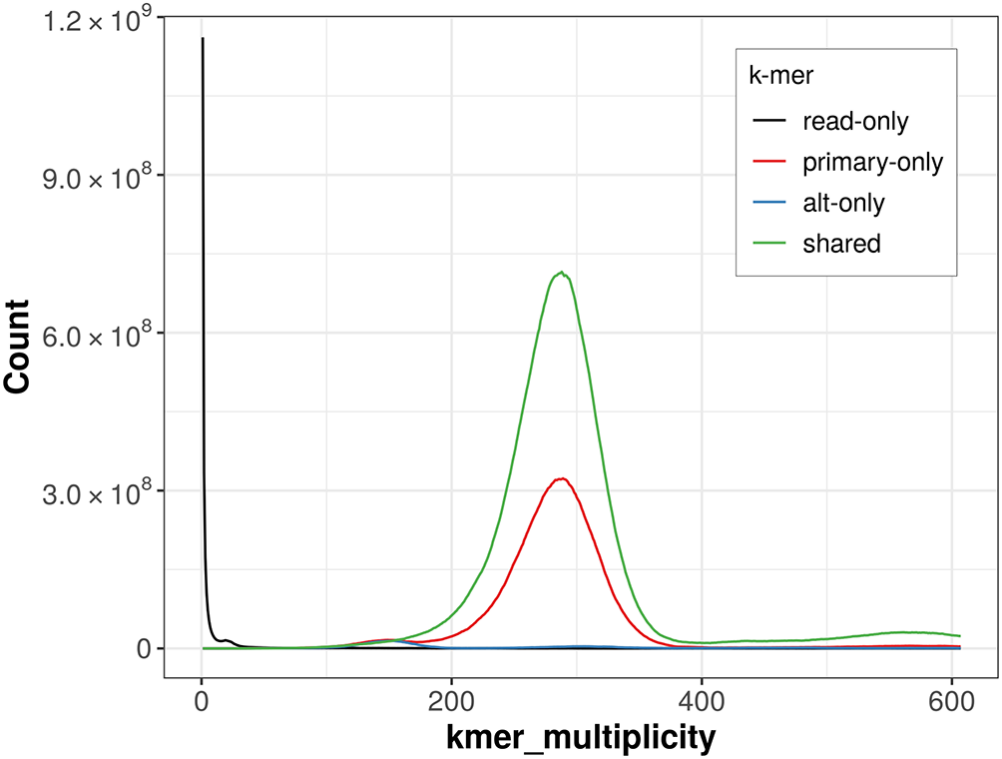
Evaluation of *k*-mer completeness using MerquryFK. This plot illustrates the recovery of *k*-mers from the original read data in the final assemblies. The horizontal axis represents *k*-mer multiplicity, and the vertical axis shows the number of *k*-mers. The black curve represents *k*-mers that appear in the reads but are not assembled. The green curve corresponds to *k*-mers shared by both haplotypes, and the red and blue curves show *k*-mers found only in one of the haplotypes.

The combined primary and alternate assemblies achieve an estimated QV of 57.4. The *k*-mer completeness is 97.64% for the primary assembly, 70.91% for the alternate haplotype, and 99.71% for the combined assemblies ( Figure 4).

**Figure 5.**
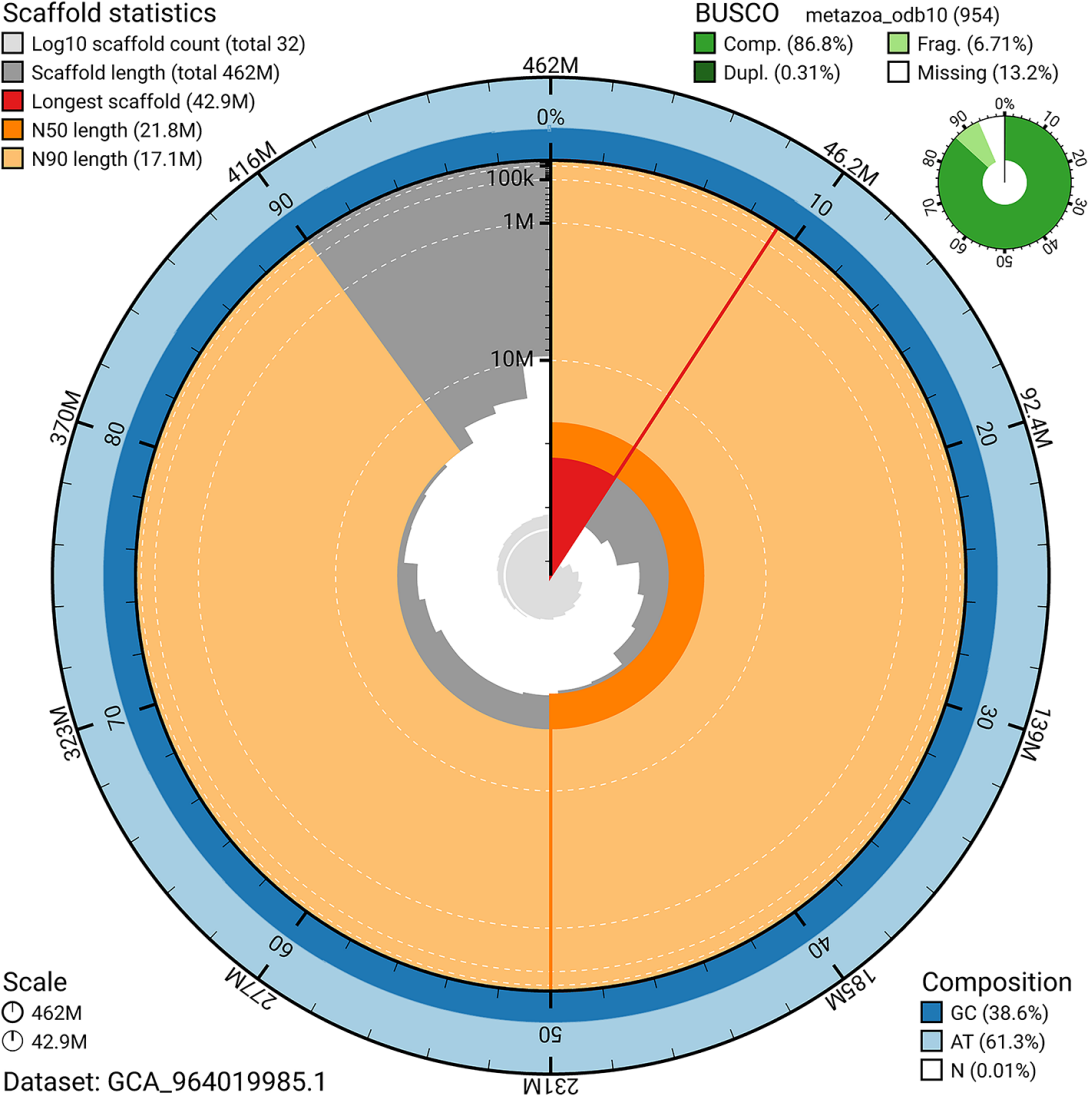
Assembly metrics for jsAurSpec1.1. The BlobToolKit snail plot provides an overview of assembly metrics and BUSCO gene completeness. The circumference represents the length of the whole genome sequence, and the main plot is divided into 1 000 bins around the circumference. The outermost blue tracks display the distribution of GC, AT, and N percentages across the bins. Scaffolds are arranged clockwise from longest to shortest and are depicted in dark grey. The longest scaffold is indicated by the red arc, and the deeper orange and pale orange arcs represent the N50 and N90 lengths. A light grey spiral at the centre shows the cumulative scaffold count on a logarithmic scale. A summary of complete, fragmented, duplicated, and missing BUSCO genes in the metazoa_odb10 set is presented at the top right. An interactive version of this figure can be accessed on the BlobToolKit viewer.

**Figure 6.**
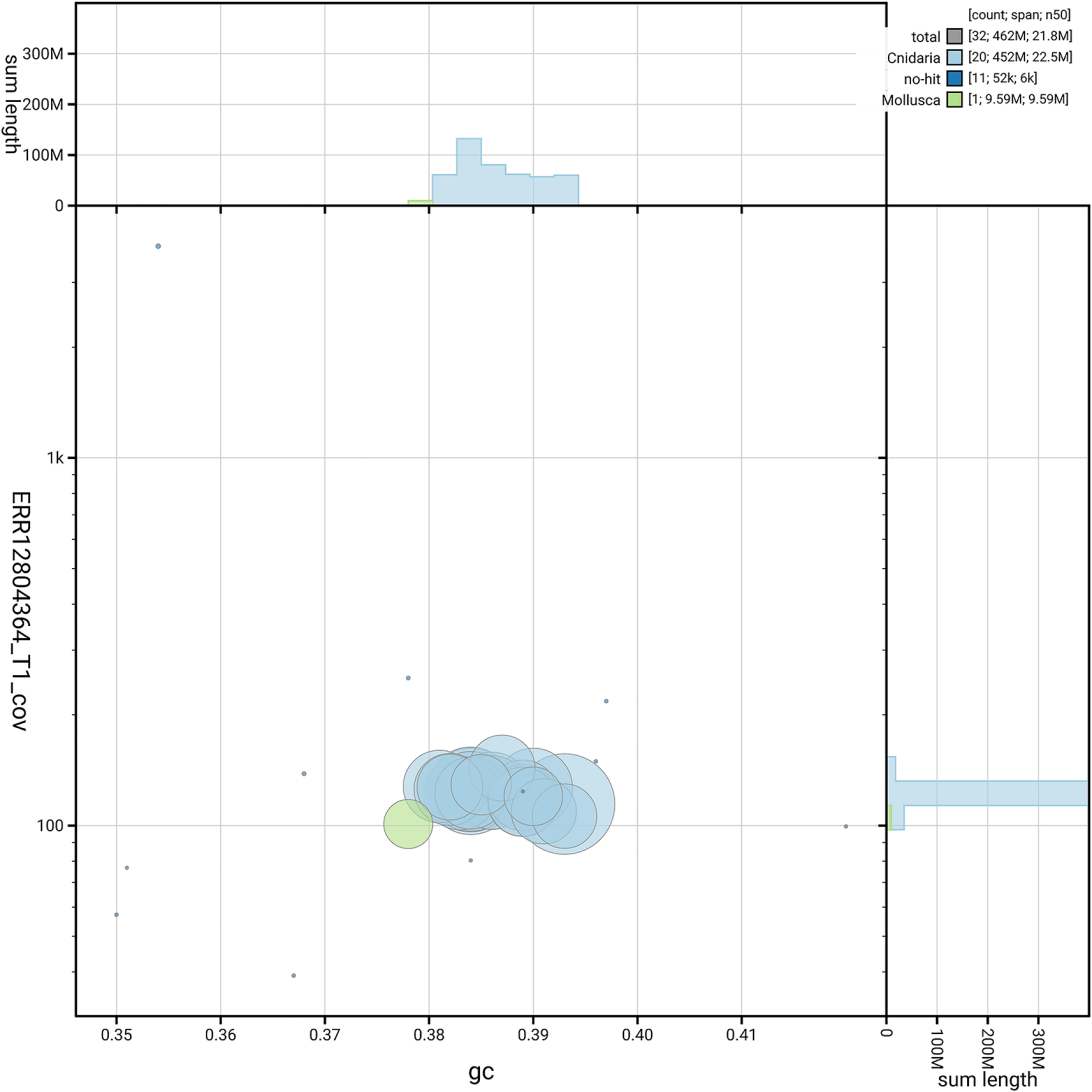
BlobToolKit GC-coverage plot for jsAurSpec1.1. Blob plot showing sequence coverage (vertical axis) and GC content (horizontal axis). The circles represent scaffolds, with the size proportional to scaffold length and the colour representing phylum membership. The histograms along the axes display the total length of sequences distributed across different levels of coverage and GC content. An interactive version of this figure is available on the BlobToolKit viewer.

BUSCO v.5.5.0 analysis using the metazoa_odb10 reference set ( *n* = 954) identified 86.8% of the expected gene set (single = 86.5%, duplicated = 0.3%). The snail plot in Figure 5 summarises the scaffold length distribution and other assembly statistics for the primary assembly. The blob plot in Figure 6 shows the distribution of scaffolds by GC proportion and coverage.

Table 4 lists the assembly metric benchmarks adapted from Rhie *et al.* (2021) and the Earth BioGenome Project Report on Assembly Standards September 2024. The EBP metric, calculated for the primary assembly, is **6.C.Q58**, meeting the recommended reference standard.

**Table 4.** Earth Biogenome Project summary metrics for the *Aurelia* sp. 4 assembly.

Measure	Value	Benchmark
EBP summary (primary)	6.C.Q58	6.C.Q40
Contig N50 length	5.47 Mb	≥ 1 Mb
Scaffold N50 length	21.79 Mb	= chromosome N50
Consensus quality (QV)	Primary: 58.9; alternate: 56.2; combined: 57.4	≥ 40
*k*-mer completeness	Primary: 97.64%; alternate: 70.91%; combined: 99.71%	≥ 95%
BUSCO	C:86.8% [S:86.5%; D:0.3%]; F:6.7%; M:6.5%; n:954	S > 90%; D < 5%
Percentage of assembly assigned to chromosomes	99.99%	≥ 90%

**Notes:** EBP summary uses log10(Contig N50); chromosome-level (C) or log10(Scaffold N50); Q (Merqury QV). BUSCO: C=complete; S=single-copy; D=duplicated; F=fragmented; M=missing; n=orthologues.

## Metagenome report

We recovered three bins from the metagenome assembly, of which two met the criteria for MAGs, including one fully circularised genome. The recovered bins represented two bacterial phyla. Mean completeness was 89.1% with 2.3% contamination. Table 5 summarises the taxa and quality of the metagenome bins.

**Table 5.** Quality metrics and taxonomic assignments of the binned metagenomes.

NCBI taxon	Taxid	GTDB taxonomy	Quality	Size (bp)	Contigs	Circular	Mean coverage	Completeness (%)	Contamination (%)
Mycoplasmataceae bacterium	2685873	Bacilli	Medium	817 844	10	Yes	15.35	96.62	0
Mycoplasmatales bacterium	2023991	Bacilli	High	407 015	1	Yes	8.83	72.68	2.26
Endozoicomonas sp.	1892382	Gammaproteobacteria	High	8 194 073	52	No	21.46	98.06	4.57

Software tool versions and sources are given in Table 6.

**Table 6.** Software versions and sources.

Software	Version	Source
**BEDTools**	2.30.0	https://github.com/arq5x/bedtools2
**bin3C**	0.3.3	https://github.com/cerebis/bin3C
**BLAST**	2.14.0	ftp://ftp.ncbi.nlm.nih.gov/blast/executables/blast+/
**BlobToolKit**	4.3.9	https://github.com/blobtoolkit/blobtoolkit
**BUSCO**	5.5.0	https://gitlab.com/ezlab/busco
**bwa-mem2**	2.2.1	https://github.com/bwa-mem2/bwa-mem2
**checkM**	2015-01-16	https://ecogenomics.github.io/CheckM/
**Cooler**	0.8.11	https://github.com/open2c/cooler
**DIAMOND**	2.1.8	https://github.com/bbuchfink/diamond
**dRep**	3.4.0	https://github.com/MrOlm/drep
**fasta_windows**	0.2.4	https://github.com/tolkit/fasta_windows
**FastK**	1.1	https://github.com/thegenemyers/FASTK
**Gfastats**	1.3.6	https://github.com/vgl-hub/gfastats
**GenomeScope2.0**	2.0.1	https://github.com/tbenavi1/genomescope2.0
**GTDB-Tk **	1.2.1	https://github.com/Ecogenomics/GTDBTk
**Hifiasm**	0.19.8-r603	https://github.com/chhylp123/hifiasm
**HiGlass**	1.13.4	https://github.com/higlass/higlass
**MAGScoT**	1.0.0	https://github.com/ikmb/MAGScoT
**MaxBin**	2.2.7	https://sourceforge.net/projects/maxbin/
**MerquryFK**	1.1.2	https://github.com/thegenemyers/MERQURY.FK
**MetaBAT2**	2.15-15-gd6ea400	https://bitbucket.org/berkeleylab/metabat
**metaMDBG**	Pre-release	https://github.com/GaetanBenoitDev/metaMDBG
**metaTOR**	Pre-release	https://github.com/koszullab/metaTOR
**Minimap2**	2.24-r1122	https://github.com/lh3/minimap2
**MitoHiFi**	2	https://github.com/marcelauliano/MitoHiFi
**MultiQC**	1.14; 1.17 and 1.18	https://github.com/MultiQC/MultiQC
**Nextflow**	23.04.1	https://github.com/nextflow-io/nextflow
**PretextSnapshot**	0.0.5	https://github.com/sanger-tol/PretextSnapshot
**PretextView**	1.0.3	https://github.com/sanger-tol/PretextView
**Prokka**	1.14.5	https://github.com/tseemann/prokka
**Seqtk**	1.3	https://github.com/lh3/seqtk
**Singularity**	3.9.0	https://github.com/sylabs/singularity
**sanger-tol/ascc**	0.1.0	https://github.com/sanger-tol/ascc
**sanger-tol/blobtoolkit**	0.4.0	https://github.com/sanger-tol/blobtoolkit
**sanger-tol/curationpretext**	1.4.2	https://github.com/sanger-tol/curationpretext
**TreeVal**	1.4.0	https://github.com/sanger-tol/treeval
**YaHS**	1.1a.2	https://github.com/c-zhou/yahs

### Wellcome Sanger Institute – Legal and Governance

The materials that have contributed to this genome note have been supplied by a Tree of Life collaborator. The Wellcome Sanger Institute employs a process whereby due diligence is carried out proportionate to the nature of the materials themselves, and the circumstances under which they have been/are to be collected and provided for use. The purpose of this is to address and mitigate any potential legal and/or ethical implications of receipt and use of the materials as part of the research project, and to ensure that in doing so we align with best practice wherever possible.

The overarching areas of consideration are:

• Ethical review of provenance and sourcing of the material
• Legality of collection, transfer and use (national and international)

Each transfer of samples is undertaken according to a Research Collaboration Agreement or Material Transfer Agreement entered into by the Tree of Life collaborator, Genome Research Limited (operating as the Wellcome Sanger Institute) and in some circumstances other Tree of Life collaborators.

## Data Availability

European Nucleotide Archive: Aurelia sp. 4 Dawson *et al.*, 2005 (moon jellyfish). Accession number PRJEB74055. The genome sequence is released openly for reuse. The *Aurelia* sp. 4 genome sequencing initiative is part of the Aquatic Symbiosis Genomics Project (PRJEB43743) and Sanger Institute Tree of Life Programme (PRJEB43745). All raw sequence data and the assembly have been deposited in INSDC databases. The genome will be annotated using available RNA-Seq data and presented through the Ensembl pipeline at the European Bioinformatics Institute. Raw data and assembly accession identifiers are reported in Table 1 and Table 2. Production code used in genome assembly at the WSI Tree of Life is available at https://github.com/sanger-tol . Table 6 lists software versions used in this study. Contributors are listed at the following links:
• Members of the Wellcome Sanger Institute Tree of Life Management, Samples and Laboratory team
• Members of Wellcome Sanger Institute Scientific Operations – Sequencing Operations
• Members of the Wellcome Sanger Institute Tree of Life Core Informatics team
• Members of the EBI Aquatic Symbiosis Genomics Data Portal Team
• The Aquatic Symbiosis Genomics Project leadership
