# Supplementary material for: The chromosomal genome sequence of the moon jellyfish, Aurelia sp. 4 Dawson et al. 2005 (Semaeostomeae: Ulmaridae) and its associated microbial metagenome sequences
Source: Wellcome Open Res. 2026 Mar 25;11:189. [Version 1] doi: 10.12688/wellcomeopenres.25907.1 (PMC13157608; doi:10.12688/wellcomeopenres.25907.1)
Supplement: Peer review history

# Reviewer report: Reviewer response for version 1

**DOI:** 10.21956/wellcomeopenres.28516.r154029

**Published:** 2026-05-20

**Authors:** Adrián Jaimes-Becerra

**Affiliations:** 1 Tohoku University, Sendai, Japan

This manuscript presents a chromosome-level genome assembly for *Aurelia* sp. 4 together with associated microbial metagenome assemblies. The study provides a valuable genomic resource for Scyphozoa and the broader cnidarian research community. The genome assembly itself is of high quality, the manuscript is generally well organized, the figures are informative and appropriate, and the biological context is presented. In particular, the authors clearly explain the significance of *Aurelia* sp. 4 as a non-photosymbiotic species within the Aquatic Symbiosis Genomics Project.

 However, despite these strengths, some aspects could be improved to strengthen reproducibility and interpretation:

 1. Genome size discrepancy: The GenomeScope analysis estimated a haploid genome size of approximately 207.7 Mb, whereas the final assembly size is reported as 462.1 Mb. The manuscript would benefit from a clearer discussion of this discrepancy and whether it may reflect assembly-related factors.

 2. Relatively low BUSCO completeness: Although the reported BUSCO completeness of 86.8% is acceptable, it appears somewhat lower than expected for a chromosome-level assembly of this quality. Additional discussion regarding potential causes of the missing BUSCOs would strengthen the interpretation of assembly completeness.

 3. Incomplete methodological reproducibility: While the methods section is extensive overall, several parameters and filtering criteria are insufficiently described, which may limit reproducibility. For example, read filtering thresholds are not specified, detailed hifiasm parameters are absent, and the cutoff criteria used in purge_dups are not described. If default parameters were used, this should be stated.

Are sufficient details of methods and materials provided to allow replication by others?

Partly

Is the rationale for creating the dataset(s) clearly described?

Yes

Are the datasets clearly presented in a useable and accessible format?

Yes

Are the protocols appropriate and is the work technically sound?

Yes

Reviewer Expertise:

Evolutionary biology, molecular biology, bioinformatics.

I confirm that I have read this submission and believe that I have an appropriate level of expertise to confirm that it is of an acceptable scientific standard, however I have significant reservations, as outlined above.

# Reviewer report: Reviewer response for version 1

**DOI:** 10.21956/wellcomeopenres.28516.r154028

**Published:** 2026-05-09

**Authors:** Bingpeng Xing

**Affiliations:** 1 Third Institute of Oceanography Ministry of Natural Resources, Xiamen, Fujian, China

This manuscript reports a chromosome-level reference genome for *Aurelia* sp. 4, together with associated microbial metagenome assemblies. Overall, the dataset is valuable. The sequencing depth is substantial, and the study uses an appropriate combination of PacBio HiFi, Hi-C, and RNA-seq data. The assembly workflow is generally sound, and the authors provide manual curation and several quality assessments. The final assembly is 462.10 Mb in length, with 99.99% of the sequence anchored to 21 chromosomal pseudomolecules. The mitochondrial genome was also assembled, and three metagenomic bins were recovered, two of which were classified by the authors as high-quality MAGs. These results make the manuscript a useful genomic resource.

 However, several points require clarification before the dataset can be fully evaluated.

 First, the GenomeScope estimates appear inconsistent with other assembly statistics. The manuscript reports a haploid genome size estimate of 207.74 Mb, heterozygosity of 50.00%, and repeat content of 31.43%, whereas the final assembly size is 462.10 Mb. The authors also state that the PacBio data provided approximately 154× coverage based on the estimated genome size. These values do not seem internally consistent and should be rechecked or clearly explained.

 Second, the BUSCO completeness is reported as 86.8%, which is below the S > 90% benchmark listed in the manuscript. However, the authors state that the assembly meets the recommended EBP reference standard. This point needs clarification. In particular, the authors should explain whether the relatively low BUSCO score reflects limited suitability of the Metazoa dataset for cnidarian genomes, or alternatively provide BUSCO results using a more appropriate Cnidaria-specific or related lineage dataset.

 Third, the basis for species identification remains insufficiently described. The manuscript states that the specimen was identified by Michael Dawson, but it does not clearly explain which morphological or molecular evidence was used to confirm the individual as *Aurelia* sp. 4. Given the presence of cryptic diversity and strong morphological similarity within *Aurelia*, the authors should provide additional identification evidence, such as COI, 16S, ITS, or other diagnostic markers used in previous studies.

 Finally, as a Data Note, the manuscript would benefit from a brief “Limitations” section. This section should state clearly that the study is based on a single individual, does not include genome annotation or phylogenomic/comparative genomic analyses, and that the metagenomic results should not be interpreted as representing the overall microbiome of the species.

Are sufficient details of methods and materials provided to allow replication by others?

Partly

Is the rationale for creating the dataset(s) clearly described?

Yes

Are the datasets clearly presented in a useable and accessible format?

Yes

Are the protocols appropriate and is the work technically sound?

Partly

Reviewer Expertise:

genome，marine biodiversity，marine biology，DNA barcoding，phylogenetic analysis

I confirm that I have read this submission and believe that I have an appropriate level of expertise to confirm that it is of an acceptable scientific standard.

# Reviewer report: Reviewer response for version 1

**DOI:** 10.21956/wellcomeopenres.28516.r154037

**Published:** 2026-05-09

**Authors:** Yichen Dai

**Affiliations:** 1 Fudan University, Shanghai, China

This Data Note presents a chromosome-scale genome assembly for *Aurelia* sp. 4, a jellyfish from marine lakes in Palau, along with three metagenome-assembled genomes (MAGs) from its microbiome. The host assembly is built using a robust Tree of Life pipeline and achieves excellent contiguity and completeness. The dataset is a valuable addition to the growing genomic resources for scyphozoans, particularly given the taxon's role in the Aquatic Symbiosis Genomics project and the unique ecological context of marine lake populations.

 However, the manuscript has several weaknesses that should be addressed before it can be considered fully scientifically sound:

**1. Why sequence MAGs?**

 The Background provides a clear rationale for sequencing this species as a non‑photosymbiotic contrast. Yet there is ambiguity in the framing of the metagenome section. It is unclear whether the MAGs are presented as a simple by‑product of the sequencing effort, a biologically meaningful first look at the *Aurelia* microbiome, or a data resource for future studies. The MAGs should be described strictly as a resource and the rationale for their inclusion briefly clarified.

**2. GenomeScope2 results**

 The host assembly is of high quality. A scaffold N50 of 21.79 Mb, assignment of 99.99% of the assembly to 21 chromosomal-level scaffolds, a k‑mer completeness of 97.64% for the primary assembly, and a BUSCO score of 86.8% (with low duplication) all indicate a high‑quality reference. The mitochondrial genome is also assembled. These metrics meet the Earth BioGenome Project reference standard.

 One point, however, requires attention: GenomeScope2 estimated a haploid genome size of ~208 Mb, yet the primary assembly spans 462 Mb, more than double the estimate. The authors also mention a heterozygosity of 50%. This extreme value is unusual and likely indicates that GenomeScope could not resolve the haploid structure correctly (perhaps due to very high heterozygosity, polyploidy, or a large repeat content). The authors should discuss this discrepancy and its implications honestly. If the species is known to be diploid, the primary assembly may be a partially phased diploid representation rather than a purely haploid one, and this could explain the inflated size. Clarifying this will guide users on how to treat the assembly (e.g., whether to mask one haplotype). A short explanation, even if speculative, would improve transparency.

**3. Metagenome assembly issues**

 Only three bins were recovered, two of which are high‑quality MAGs. The methods mention that DAS Tool was used to optimise and refine bins from "each binning algorithm", yet only MetaMDBG was listed as the assembler. The metagenome assembly workflow is not fully described—were multiple binning algorithms (e.g., MaxBin, MetaBAT) run, and if so, how were their results integrated? Table 6 lists bin3C, MaxBin, MetaBAT2, and MAGScoT, but their specific roles are not detailed. A concise, step‑by‑step description of the metagenome workflow (including whether host reads were removed) is needed for reproducibility.

**Minor comments**

 1. The abstract is almost entirely assembly statistics. Consider adding a single sentence on why an *Aurelia* sp. 4 genome is of interest (e.g., as a non‑photosymbiotic contrast, marine lake endemic).

 2. In the Background section, some language is informal for a scientific paper: "Ghostly white, mostly, though other times pink (a 'blood moon'?), purplish…"and "we know from… we also know…" should be revised to a more neutral, formal style. The "blood moon" phrase is distracting and not informative.

 3. "Chromosomal pseudomolecules" may not be familiar to all readers. A brief definition or a note that these represent assembled chromosomes could help.

 4. "Nanodrop" should be "NanoDrop".

 5. The manuscript ends abruptly after the metagenome report. A short concluding paragraph that places this genome among existing *Aurelia* references, reiterates its value for symbiosis contrasts, and if appropriate, acknowledges the MAGs as a resource, would be helpful to the reader.

Are sufficient details of methods and materials provided to allow replication by others?

Yes

Is the rationale for creating the dataset(s) clearly described?

Yes

Are the datasets clearly presented in a useable and accessible format?

Yes

Are the protocols appropriate and is the work technically sound?

Yes

Reviewer Expertise:

Single-cell sequencing, genome sequencing, animal evolution

I confirm that I have read this submission and believe that I have an appropriate level of expertise to confirm that it is of an acceptable scientific standard, however I have significant reservations, as outlined above.

# Reviewer report: Reviewer response for version 1

**DOI:** 10.21956/wellcomeopenres.28516.r153299

**Published:** 2026-05-06

**Authors:** Andrea M. Quattrini

**Affiliations:** 1 Smithsonian Institution National Museum of Natural History, Washington, DC, USA

**Review – Jellyfish Genome Note**

 This manuscript presents a high-quality chromosomal-level genome assembly for *Aurelia* sp. 4, a member of a taxonomically challenging and ecologically important genus of scyphozoan jellyfish. The assembly is well executed, with strong coverage and high contiguity. The inclusion of a mitochondrial genome is appropriate, and the recovery of metagenome-assembled genomes (MAGs) could add value. Given the ongoing need for genomic resources in cnidarians, particularly non-model scyphozoans, this dataset represents a useful contribution.

 However, the manuscript would benefit from a clearer statement of its objectives. At present, it is not explicitly framed as a data resource paper, and this lack of framing creates confusion, particularly regarding the inclusion of MAGs. If the primary goal is to generate and release a high-quality reference genome, this should be stated clearly at the outset and reinforced throughout.

 Related to this, the inclusion of MAGs is not well justified. It is unclear whether these represent biologically meaningful components of the *Aurelia* microbiome, incidental recoveries from sequencing data, or exploratory analyses. The manuscript should clarify why these MAGs are included and how they relate to the overall objectives. If no biological interpretation is intended, this section should be explicitly framed as a resource rather than as a result with implied ecological significance.

 The GenomeScope estimate of ~207 Mb differs substantially from the final assembly size (~462 Mb), and the reported heterozygosity (50%) is unusually high. This discrepancy should be addressed more explicitly, as it has implications for how the assembly is interpreted.

 RNA sequencing data were generated, but no annotation is presented. The authors should either justify why annotation was not included. Expand this with a few more sentences.

 Finally, the manuscript would benefit from a short summary or concluding section. Even for a genome note, a brief synthesis placing this assembly in the context of other cnidarian genomes (e.g., genome size, structure, completeness) would improve the paper. The current ending is abrupt. If MAGs are retained, their relevance should also be briefly revisited in this section. Alternatively, if the manuscript is intended strictly as a data resource, this should be clearly stated and consistently reflected in the scope of the paper.

 Minor Comments

 Abstract

 Consider briefly stating why *Aurelia* sp. 4 is of particular interest, rather than focusing solely on assembly statistics.

 Terminology

 “Chromosomal pseudomolecule” may be familiar to some readers, but a brief definition would improve clarity.

 Background

- “Eponymous celestial body” reads as informal—consider revising.
- “Blood moon?” appears stylistically out of place; revise or remove.
- Avoid phrasing such as “we know”; rephrase more formally.

The discussion of the absence of zooxanthellae is interesting but should be more clearly linked to the stated goals of the Aquatic Symbiosis Genomics project. In particular, the manuscript would benefit from a clearer statement of purpose regarding symbiosis.

 More generally, the objectives of the study should be stated explicitly, including justification for the inclusion of MAGs, which currently lack background or context.

 Methods and Results

 Overall, the methods are detailed and reproducible. However, some sections could be streamlined (e.g., repeated quantification steps) to improve readability.

- **Sample acquisition**: Remove “The same specimen was used for RNA sequencing.”
- Ensure consistent use of terms such as “chromosomal pseudomolecules,” “scaffolds,” and “primary assembly.” Would definitions help?
- Include a brief clarification of how RNA data are intended to be used, as this currently feels underdeveloped.
- Only three MAGs were recovered; include a few sentences clarifying their relevance or rationale for inclusion.

Are sufficient details of methods and materials provided to allow replication by others?

Yes

Is the rationale for creating the dataset(s) clearly described?

Yes

Are the datasets clearly presented in a useable and accessible format?

Yes

Are the protocols appropriate and is the work technically sound?

Yes

Reviewer Expertise:

Cnidarian evolution, phylogenomics, population genetics

I confirm that I have read this submission and believe that I have an appropriate level of expertise to confirm that it is of an acceptable scientific standard, however I have significant reservations, as outlined above.
